# Cardiovascular imaging research priorities

**DOI:** 10.1136/openhrt-2023-002378

**Published:** 2023-08-16

**Authors:** Jacqueline Ann Langdon MacArthur, Guo Liang Yong, Marc R Dweck, Timothy A Fairbairn, Jonathan Weir-McCall, Esther Puyol-Antón, Julian Meldrum, Phillip Blakelock, Samaira Khan, Lynn Morrice, Cathie L M Sudlow, Michelle C Williams

**Affiliations:** 1 British Heart Foundation Data Science Centre, Health Data Research UK, London, UK; 2 Centre for Cardiovascular Science, University of Edinburgh, Edinburgh, UK; 3 Liverpool Centre for Cardiovascular Science, Liverpool Heart and Chest Hospital, Liverpool, UK; 4 School of Clinical Medicine, University of Cambridge, Cambridge, UK; 5 Department of Radiology, Royal Papworth Hospital, Cambridge, UK; 6 School of Biomedical Engineering and Imaging Sciences, King's College London, London, UK

**Keywords:** diagnostic imaging, research design, health services

## Abstract

**Objectives:**

Two interlinked surveys were organised by the British Heart Foundation Data Science Centre, which aimed to establish national priorities for cardiovascular imaging research.

**Methods:**

First a single time point public survey explored their views of cardiovascular imaging research. Subsequently, a three-phase modified Delphi prioritisation exercise was performed by researchers and healthcare professionals. Research questions were submitted by a diverse range of stakeholders to the question ‘What are the most important research questions that cardiovascular imaging should be used to address?’. Of these, 100 research questions were prioritised based on their positive impact for patients. The 32 highest rated questions were further prioritised based on three domains: positive impact for patients, potential to reduce inequalities in healthcare and ability to be implemented into UK healthcare practice in a timely manner.

**Results:**

The public survey was completed by 354 individuals, with the highest rated areas relating to improving treatment, quality of life and diagnosis. In the second survey, 506 research questions were submitted by diverse stakeholders. Prioritisation was performed by 90 researchers or healthcare professionals in the first round and 64 in the second round. The highest rated questions were ‘How do we ensure patients have equal access to cardiovascular imaging when it is needed?’ and ‘How can we use cardiovascular imaging to avoid invasive procedures’. There was general agreement between healthcare professionals and researchers regarding priorities for the positive impact for patients and least agreement for their ability to be implemented into UK healthcare practice in a timely manner. There was broad overlap between the prioritised research questions and the results of the public survey.

**Conclusions:**

We have identified priorities for cardiovascular imaging research, incorporating the views of diverse stakeholders. These priorities will be useful for researchers, funders and other organisations planning future research.

**WHAT IS ALREADY KNOWN ON THIS TOPIC**

- Cardiovascular imaging research has enormous potential to improve health, but national priorities for research in this area from a diverse range of stakeholders have not been established.

**WHAT THIS STUDY ADDS**

- In a survey of patients and the public, we have established their views on the most important areas of cardiovascular research and how research should be prioritised.
- In a multiphase modified Delphi prioritisation exercise performed by researchers and healthcare professionals, we have established national priorities for cardiovascular imaging research.

**HOW THIS STUDY MIGHT AFFECT RESEARCH, PRACTICE OR POLICY**

- This information will impact choices regarding the design of cardiovascular research studies and ensure that views of patients and public are included.

## Introduction

Cardiovascular disease remains the leading cause of death globally,^1^ with 7.6 million people living with heart and circulatory diseases in the UK.^2^ The use of cardiovascular imaging in patient care has increased rapidly, with an annualised growth rate of 6.6% for cardiac examinations.^3^ Cardiovascular imaging is now central to the diagnosis and management of patients with cardiovascular disease. However, priorities for future research in this area have not been established.

Cardiovascular imaging research has enormous potential to improve cardiovascular health through improvements in disease phenotyping, diagnosis and risk assessment, the development of new imaging and image analysis techniques to guide patient management and the use of imaging markers as endpoints in clinical trials. Linkage of images to other healthcare data could improve study design by integrating imaging information with cardiovascular outcomes, comorbidities, behavioural measures and medication use. Artificial intelligence and machine learning may be of added benefit in this area, for example, through improved ‘personalised’ risk stratification or more rapid and detailed image assessment.

Previous efforts to develop research priorities in cardiovascular imaging established a list of the top 10 research priorities from a consensus survey.^4^ This prioritised submitted questions which were primarily focused on CT and priority themes included questions involving coronary artery disease, image protocol optimisation and choice of imaging strategy. However, this survey involved a limited range of stakeholders due to the methodology of involving members of a single professional society, focused primarily on one imaging modality and only assessed a single prioritisation domain, the clinical importance of the research question.^4^ Importantly, the views of patients were not assessed. There, therefore, remains an important need for the development of prioritised research questions based on the input of a wide range of stakeholders, including patients and the public, and reflecting an approach which is not specific for individual imaging modalities.

The British Heart Foundation (BHF) Data Science Centre aims to improve cardiovascular health through enabling research that integrates interdisciplinary expertise in data science with cardiovascular health-relevant data from across the UK and beyond. To inform the focus of our activities and to assist others considering research in this area, we sought to explore the views of imagers, researchers, patients and the public on how research should be prioritised and then to establish national priorities for cardiovascular imaging research using a consensus survey method involving a diverse range of stakeholders.

## Methods

**Figure 1:**
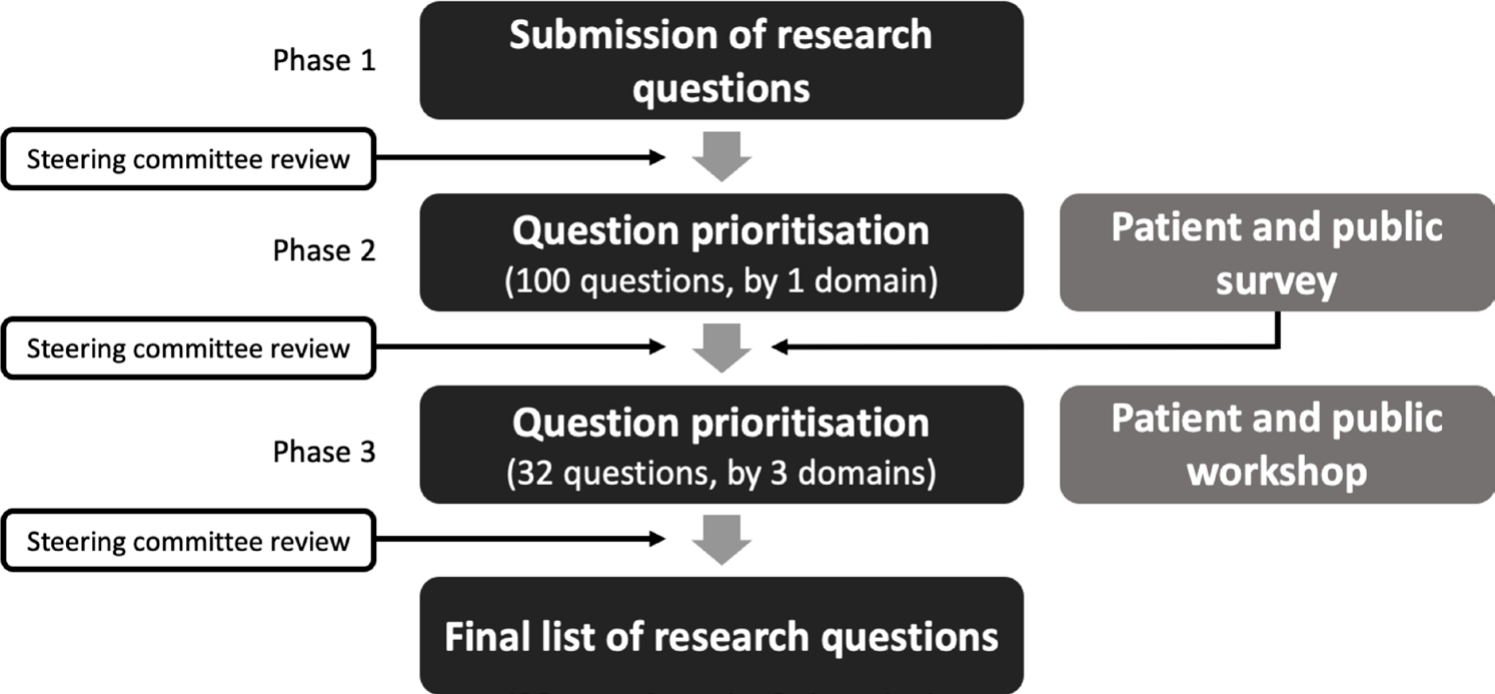
Prioritisation exercise methodology.

Two interlinked surveys were performed in order to establish national priorities for cardiovascular imaging research. First, a single time point survey of patients and the public was performed to ascertain what areas of cardiovascular research they thought were most important and how research should be prioritised. Second, a prioritisation exercise was performed by researchers and healthcare professionals using a modified Delphi technique (figure 1) to gather and prioritise cardiovascular imaging research questions. The prioritisation exercise methods were based on previous work in this area and adapted by the steering committee to meet the study needs. The first phase involved gathering research questions from a diverse range of stakeholders, including patients, researchers, healthcare professionals and members of the public. This was followed by two rounds of prioritisation guided by this patient and public consultation exercise.

A steering committee (MCW, JALM, MRD, TAF, JW-M, EP-A, JM, PB and GLY) was formed, composed of the study management team from the BHF Data Science Centre, researchers representing the fields of cardiovascular imaging and image analysis and two patient/public representatives. The steering committee reviewed the methodology, selected the domains for prioritisation and selected the questions to take forwards in each round of prioritisation.

### Patient and public involvement

We involved members of the BHF Data Science Centre Public Advisory Group in the development, design, management, analysis and dissemination of this research. The group comprised individuals with diverse backgrounds, experiences and interests in the field of data science. The survey designed for the patients and the general public was codesigned with our group of public representatives. This inclusive process ensured that the survey was accessible, comprehensible and written in plain English, with careful consideration given to the clarity and inclusivity of all questions and response options. In addition, the steering committee for the project included two patient/public representatives.

### Phase 1: patient and public survey

The survey for patients and members of the public included two main questions, additional questions regarding basic demographic information and free text comment boxes. The first main question asked participants to, ‘Help us identify which areas of research are most important to patients and the public’. Participants were provided with a list of 10 types of cardiovascular disease research and were asked to rate these from 1 (not at all important) to 5 (very important). The second main question asked, ‘There are lots of different ways that are currently used to assess which research studies should be funded and carried out. Which of the following are important to you?’. Participants were presented with 13 ways of assessing research studies and asked to score each from 1 (not at all important) to 5 (very important). The survey was carried out using an online tool (SurveyMonkey, https://www.surveymonkey.com) and was open for participation for 1 month. The survey was widely communicated across public engagement networks and social media to ensure as broad a range of participants as possible.

### Phase 1: prioritisation exercise

In phase 1 of the Delphi process, cardiovascular imaging research questions were gathered from a wide range of stakeholders to answer the question ‘What are the most important research questions that cardiovascular imaging should be used to address?’. Answers to this question were submitted as part of a cardiovascular imaging workshop organised by the BHF Data Science Centre (number of participants=86)^5^ as well as through an online survey form (SurveyMonkey, https://www.surveymonkey.com, number of submissions=442). To obtain suggested research questions from as wide a range of stakeholders as possible this survey was advertised and distributed widely, including through national cardiovascular and cardiovascular imaging societies, patient networks (including HDR UK Voices, https://www.hdruk.ac.uk/about-us/involving-and-engaging-patients-and-the-public/get-involved/join-hdr-uk-voices/), a website news article, and through social media (LinkedIn, Twitter, YouTube). The survey remained open for 3 weeks and there was no limit to the number of questions that respondents could submit.

### Phase 2: prioritisation exercise

The submitted research questions were reviewed by the steering committee and agreed by consensus. Questions were excluded if they were duplicates, would not be possible to address or were not relevant to the primary question. Where feasible, questions that covered the same subject were combined. References to specific modalities were removed where possible to create modality agnostic research questions. Questions were also edited as needed for clarity. Following this process, 100 questions were selected to take forwards for prioritisation (online supplemental table 1).

10.1136/openhrt-2023-002378.supp1 Supplementary data

Survey participants for phase 2 consisted of healthcare professionals, cardiovascular imaging researchers, data science and/or computer science researchers, and researchers from other relevant disciplines. They were identified through an open call for participants which was advertised via email to previous BHF Data Science Centre workshop participants (number of participants=86), professional cardiovascular and cardiovascular imaging societies, and social media (LinkedIn, Twitter). Participants were asked to select the one stakeholder category which best represented them.

Participants rated each question on their perception of its potential for positive impact for patients, from 1, lowest priority, to 9, highest priority. Questions were presented to participants with pages of questions in a random order. Dedicated online software was used for this prioritisation (DelphiManager, https://www.comet-initiative.org/delphimanager/) and the prioritisation website remained open for 2 months.

### Phase 3: prioritisation exercise

The steering committee reviewed the results of the phase 2 prioritisation exercise, including the highest-rated questions from all participants and from each of the stakeholder groups. This information was used to select questions for further prioritisation, ensuring that the highest-rated questions from each stakeholder group were included. Further editing of these questions was performed to combine relevant themes and to ensure clarity. This resulted in 32 questions for further prioritisation (online supplemental table 2).

Three domains were selected for this round of prioritisation, including the original domain, positive impact for patients and two new domains. The choice of domains was informed by the results of the phase 1 public survey, as well as by steering committee discussion. Based on this information, the two additional domains selected were ‘potential to reduce inequalities in healthcare’ and ‘ability to be implemented into UK healthcare practice in a timely manner’. All 32 questions were scored separately for each of the three domains.

Individuals who participated in the phase 2 survey were invited to participate in the phase 3 survey. This second round of prioritisation was performed using an online survey form (SurveyMonkey, https://www.surveymonkey.com) due to the requirement to perform prioritisation on multiple domains.

The results of this round of prioritisation were used to create the final ordered list of research priorities for each of the prioritisation domains, for all participants and for each of the stakeholder groups.

### Phase 4: patient and public workshop

Once the results of the surveys were available, we discussed these with our BHF Data Science Centre Public Advisory Group in a workshop attended by 15 public contributors in order to gather their views on the results and future actions.

### Statistical analysis

Statistical analysis was performed using R (V.4.0.3, R Foundation for Statistical Computing, Vienna, Austria) and Microsoft Excel for Mac (V.16.69). Means±SDs were used to summarise ratings for each question. The research questions with the highest mean rating were deemed to be the highest priority. Analysis was performed for one prioritisation domain in phase 2 (positive impact for patients) and three prioritisation domains in phase 3 (positive impact for patients, potential to reduce inequalities in healthcare, ability to be implemented into UK healthcare practice in a timely manner). Results are presented for all participants and stratified by the four stakeholder groups (healthcare professionals, cardiovascular imaging researchers, (data science) and/or computer science researchers, and researchers from other relevant disciplines).

## Results

### Phase 1: patient and public survey

**Figure 2:**
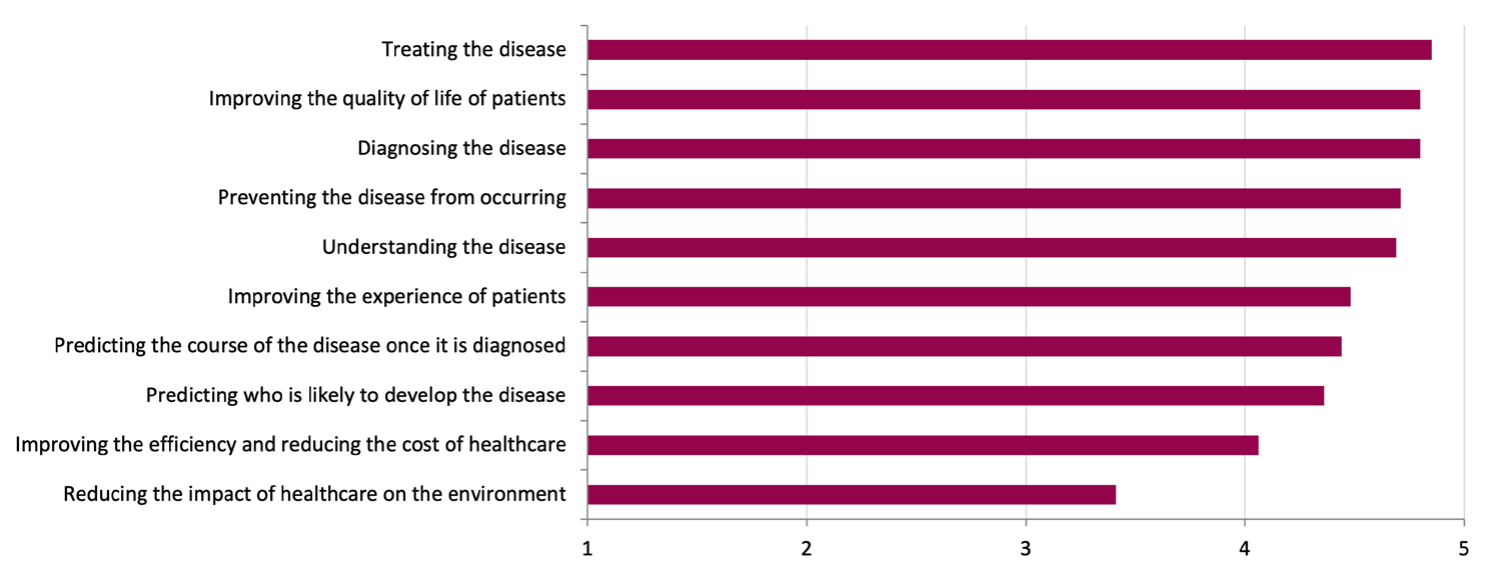
Results of the public survey showing which areas of research were most important to patients and the public. Bar chart showing weighted average of the submitted survey responses.

**Figure 3:**
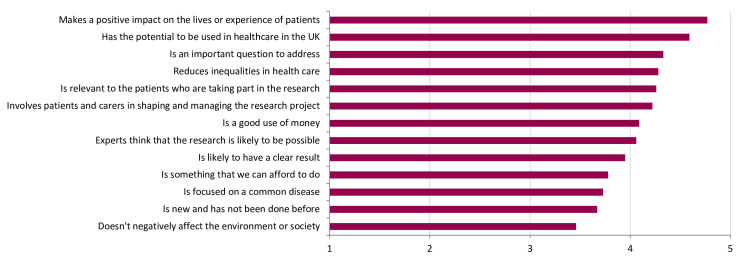
Results of the public survey showing how patients and the public thought that research should be prioritised. Bar chart showing weighted average of the submitted survey responses.

There were 354 patient and public respondents who completed the survey from the UK. Of these, 59% (n=209/354) were aged 55 or over, 66% (n=231/352) were female and 4% (n=15/351) were not of white ethnicity (online supplemental figure 1). Most participants (83%, n=293/353) had, or knew someone with, cardiovascular disease. The highest rated areas of research were ‘treating the disease’ (mean rating 4.9±0.4), ‘improving the quality of life of patients’ (mean rating 4.8±0.5) and ‘diagnosing the disease’ (mean rating 4.8±0.6, figure 2). The highest rated ways of prioritising research were ‘makes a positive impact on the lives or experience of patients’ (mean rating 4.8±0.6), ‘has the potential to be used in healthcare in the UK’ (mean rating 4.6±0.7) and ‘is an important question to address’ (mean rating 4.3±0.8) and ‘reduces inequalities in healthcare’ (mean rating 4.3±1.0, figure 3).

### Phase 1: gathering research questions

In total 506 questions were submitted during the open call for questions and via the BHF Data Science Centre workshop. Questions were submitted by imaging, cardiovascular data science, computer science and other researchers, healthcare professionals, patients and members of the public, representatives from National Health Service organisations, representatives from relevant professional organisations, representatives from relevant companies, health data custodians and other interested individuals.

Most of the questions submitted were not modality specific (91%, n=459/506). A small number of modality specific questions were submitted, involving CT (2%, n=11/506), nuclear imaging (2%, n=11/506), MRI (1%, n=7/506), multimodality imaging (1%, n=5/506), echocardiography (1%, n=4/506), electrocardiography (0.2%, n=1/506), invasive imaging (0.2%, n=1/506), retinal imaging (0.2%, n=1/506) and X-ray imaging (0.2%, n=1/506).

**Figure 4:**
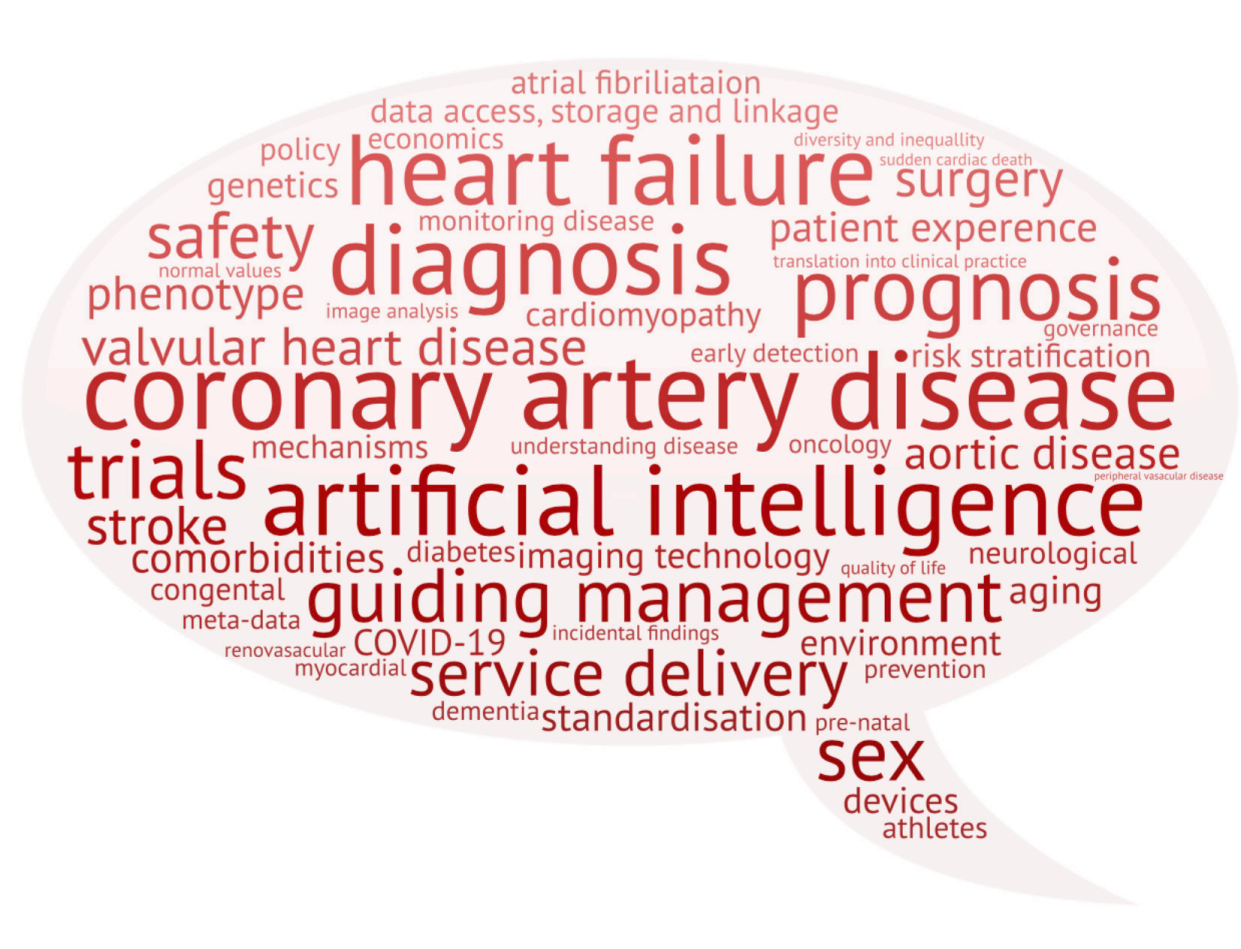
Word cloud demonstrating the range of topics covered in the submitted questions in phase 1.

The questions submitted covered a wide range of themes (figure 4), including specific conditions (eg, coronary artery disease, heart failure), aims of imaging (eg, diagnosis, guiding management), imaging data science research challenges (eg, data access, storage and linkage), computational analytical challenges (eg, artificial intelligence, image analysis) and patient factors (eg, communication, experience and access).

### Phase 2: first round of prioritisation

The steering committee selected 100 questions for prioritisation. Of the 90 participants who participated in this round of prioritisation, there were 51 healthcare professionals (57%), 24 cardiovascular imaging researchers (27%), 8 data science and/or computer science researchers (9%), and 7 researchers from other relevant disciplines (8%). The list of 100 questions ordered by their rating by all participants can be found in online supplemental table 1. The highest rated questions were ‘how can we use cardiovascular imaging to avoid invasive procedures?’ with a mean rating of 7.3±1.7, ‘can cardiovascular imaging be used to identify vulnerable atherosclerotic plaques which could cause subsequent myocardial infarction?’ (mean rating 7.3±1.6), ‘can we use cardiac imaging to predict which patients with heart failure would benefit from different treatments?’ (mean rating 7.2±1.3), ‘how can cardiovascular imaging be used to make more rapid and accurate diagnoses?’ (mean rating 7.1±1.7) and ‘how do we use cardiovascular imaging to screen for coronary artery disease, guide management and prevent subsequent cardiovascular events?’ (mean rating 7.1±1.7).

### Phase 3: second round of prioritisation

**Figure 5:**
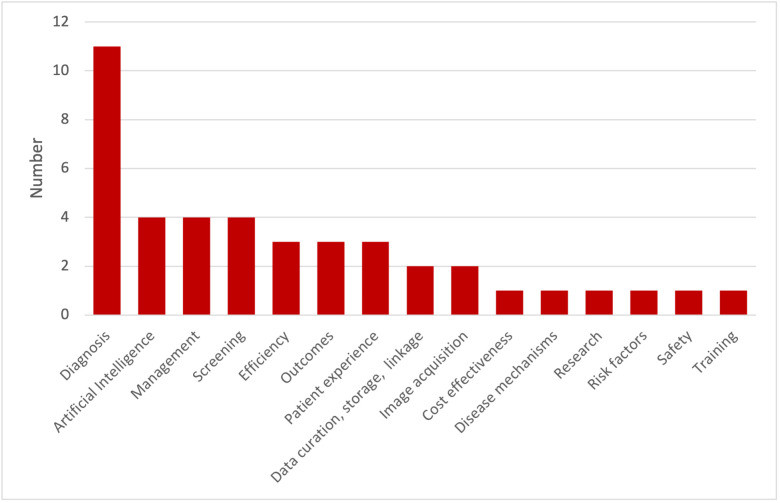
Research themes for the 32 research questions selected for prioritisation in phase 3 (questions may include more than one theme).

Based on the results of the first round of prioritisation, the steering committee selected 32 questions for prioritisation in this second round. These questions encompassed a wide range of research themes (figure 5) and cardiovascular diseases, including coronary artery disease, heart failure, cardiomyopathies, sudden cardiac death, valvular heart diseases, atrial fibrillation, aortic disease, vascular dementia, cardio-oncology, autonomic dysfunction, comorbidities, infection, inflammation and changes in the cardiovascular system associated with ageing.

There were 64 participants for this round of prioritisation, including 36 healthcare professionals (56%), 15 cardiovascular imaging researchers (23%), 7 data science and/or computer science researchers (11%) and 6 researchers from other relevant disciplines (9%).

The top 10 questions for each prioritisation domain are shown in table 1. The highest rated question for the prioritisation domains (1) positive impact for patients and (2) potential to reduce inequalities in healthcare, was ‘How do we ensure patients have equal access to cardiovascular imaging when it is needed?’ with mean ratings of 7.6±1.6 and 7.9±1.6, respectively. The highest rated question for the prioritisation domain (3) ability to be implemented into UK healthcare practice in a timely manner was ‘How can we use cardiovascular imaging to avoid invasive procedures?’ with a mean rating of 7.2±1.7. Question themes in these top 10 lists included imaging based topics such as diagnostic accuracy, image acquisition protocols and staff training; patient focused topics, including access to imaging, avoiding invasive procedures, efficiency in care pathways and improving the patient experience; research focused topics, including data curation, data linkage, and artificial intelligence; and disease focused topics, including coronary artery disease, sudden cardiac death and heart failure.

**Table 1:** Top 10 questions from the round 2 prioritisation based on (A) positive impact for patients, (B) potential to reduce inequalities in healthcare and (C) ability to be implemented into UK healthcare practice in a timely manner

**Rank**	**Question**	**Rating**
(A) Positive impact for patients		
1	How do we ensure patients have equal access to cardiovascular imaging when it is needed?	7.6±1.6
2	How can cardiovascular imaging be used to make more rapid and accurate diagnoses?	7.5±1.4
3	How do we use cardiovascular imaging to guide management, reduce disease progression and improve prognosis for patients with coronary artery disease?	7.3±1.4
4	How can we use cardiovascular imaging to avoid invasive procedures?	7.2±1.7
5	How do we use cardiovascular imaging to identify patients at risk of sudden cardiac death?	7.0±1.5
6	Can we use cardiovascular imaging to better diagnose the cause and subtypes of heart failure, and predict which patients with heart failure would benefit from different treatments?	7.0±1.4
7	Can we reduce the number of cardiac imaging tests that patients need during follow-up?	6.9±2.0
8	How do we use cardiovascular imaging to identify people at risk of developing heart failure?	6.9±1.4
9	How do we better train staff to perform and report cardiovascular imaging?	6.7±1.8
10	How do we link cardiovascular imaging data to other health data for example, NHS patient records in a safe, secure, and responsible manner, and manage public trust when using unconsented data	6.6±2.0
(B) Potential to reduce inequalities in healthcare		
1	How do we ensure patients have equal access to cardiovascular imaging when it is needed?	7.9±1.6
2	How can cardiovascular imaging be used to make more rapid and accurate diagnoses?	7.3±1.1
3	How can we use cardiovascular imaging to make care pathways more efficient and improve the cost effectiveness of cardiovascular imaging?	7.2±1.6
4	How do we use cardiovascular imaging to guide management, reduce disease progression and improve prognosis for patients with coronary artery disease?	7.1±1.4
5	How do we link cardiovascular imaging data to other health data for example, NHS patient records, in a safe, secure, and responsible manner, and manage public trust when using unconsented data?	7.0±1.9
6	How do we create a national, representative, large scale cardiovascular imaging research database with ground truth annotation to enable training and validation of AI techniques?	7.0±1.8
7	How do we use cardiovascular imaging to identify people at risk of developing heart failure?	6.9±1.3
8	Can we use AI to prioritise cardiovascular imaging scans for reporting and improve clinical decision-making based on cardiovascular imaging?	6.9±1.7
9	How can we simplify shorten and standardise cardiovascular image acquisition protocols for easier widespread use and reduced variability?	6.8±1.8
10	For cardiovascular imaging AI techniques, how do we identify and reduce bias improve generalisability and explain the results explainable AI?	6.8±2.2
(C) Ability to be implemented into UK healthcare practice in a timely manner		
1	How can we use cardiovascular imaging to avoid invasive procedures?	7.2±1.7
2	How can cardiovascular imaging be used to make more rapid and accurate diagnoses?	7.2±1.5
3	How can we use cardiovascular imaging to make care pathways more efficient and improve the cost effectiveness of cardiovascular imaging?	7.2±1.5
4	How do we ensure patients have equal access to cardiovascular imaging when it is needed?	7.2±1.8
5	How can we simplify, shorten, and standardise, cardiovascular image acquisition protocols for easier widespread use and reduced variability?	7.0±1.7
6	How do we use cardiovascular imaging to guide management reduce disease progression and improve prognosis for patients with coronary artery disease?	6.9±1.4
7	Can we reduce the number of cardiac imaging tests that patients need during follow-up?	6.9±2.0
8	How do we link cardiovascular imaging data to other health data, for example, NHS patient records in a safe, secure, and responsible manner, and manage public trust when using unconsented data?	6.8±2.0
9	Can we use AI to improve cardiovascular image acquisition improve image quality, reduce radiation and contrast doses, and reduce motion artefacts?	6.8±1.7
10	Can we use AI to prioritise cardiovascular imaging scans for reporting and improve clinical decision making based on cardiovascular imaging?	6.7±1.7

Mean±SD.

AI, artificial intelligence; NHS, National Health Service.

There was overlap between stakeholder groups in the top five questions for the prioritisation domain of positive impact for patients. For this domain, two questions featured in each of the top five lists for healthcare professionals, cardiovascular imaging researchers and data science and/or computer science researchers (table 2). However, there was less agreement for the domain potential to reduce inequalities in healthcare, with only one question, ‘How do we ensure patients have equal access to cardiovascular imaging when it is needed?,’ occurring in the top five for all three stakeholder groups. For this prioritisation domain, healthcare professionals rated questions about improving imaging tests higher while imaging researchers rated questions that involved artificial intelligence and the use of data higher. Agreement was lowest for the domain ability to be implemented into UK healthcare practice in a timely manner, with no questions appearing in the top five list for all stakeholder groups.

**Table 2:** Top five questions prioritised by healthcare professionals, cardiovascular researchers and data science and/or computer science researchers for the prioritisation domains (A) positive impact for patients, (B) potential to reduce inequalities in healthcare and (C) ability to be implemented into UK healthcare practice in a timely manner

	**Healthcare professionals**	**Cardiovascular imaging researchers**	**Data science and/or computer science researchers**
(A) Positive impact for patients			
1	How do we use cardiovascular imaging to guide management, reduce disease progression and improve prognosis for patients with coronary artery disease?	How can cardiovascular imaging be used to make more rapid and accurate diagnoses?	How do we ensure patients have equal access to cardiovascular imaging when it is needed?
2	How do we ensure patients have equal access to cardiovascular imaging when it is needed?	How do we ensure patients have equal access to cardiovascular imaging when it is needed?	Can we reduce the number of cardiac imaging tests that patients need during follow-up?
3	How can cardiovascular imaging be used to make more rapid and accurate diagnoses?	Can we use cardiovascular imaging to better diagnose the cause and subtypes of heart failure and predict which patients with heart failure would benefit from different treatments?	Can we use AI to prioritise cardiovascular imaging scans for reporting and improve clinical decision-making based on cardiovascular imaging?
4	Can we reduce the number of cardiac imaging tests that patients need during follow-up?	How can we use cardiovascular imaging to avoid invasive procedures?	How can we use cardiovascular imaging to avoid invasive procedures?
5	How do we use cardiovascular imaging to identify patients at risk of sudden cardiac death?	How do we better train staff to perform and report cardiovascular imaging?	How can cardiovascular imaging be used to make more rapid and accurate diagnoses?
(B) Potential to reduce inequalities in healthcare			
1	How do we ensure patients have equal access to cardiovascular imaging when it is needed?	How do we ensure patients have equal access to cardiovascular imaging when it is needed?	How can we use cardiovascular imaging to make care pathways more efficient and improve the cost-effectiveness of cardiovascular imaging?
2	How can cardiovascular imaging be used to make more rapid and accurate diagnoses?	How can we use cardiovascular imaging to make care pathways more efficient and improve the cost effectiveness of cardiovascular imaging?	How do we ensure patients have equal access to cardiovascular imaging when it is needed?
3	How do we use cardiovascular imaging to guide management, reduce disease progression and improve prognosis for patients with coronary artery disease?	For cardiovascular imaging AI techniques, how do we identify and reduce bias, improve generalisability, and explain the results (explainable AI)?	How do we create a national representative, large-scale cardiovascular imaging research database with ground truth annotation to enable training and validation of AI techniques?
4	Can we use AI to prioritise cardiovascular imaging scans for reporting and improve clinical decision-making based on cardiovascular imaging?	How do we link cardiovascular imaging data to other health data, for example, NHS patient records in a safe, secure, and responsible manner, and manage public trust when using unconsented data?	For cardiovascular imaging AI techniques, how do we identify and reduce bias, improve generalisability and explain the results explainable AI?
5	How can we simplify, shorten and standardise cardiovascular image acquisition protocols for easier widespread use and reduced variability?	How can cardiovascular imaging be used to make more rapid and accurate diagnoses?	How can we use cardiovascular imaging to avoid invasive procedures?
(C) Ability to be implemented into UK healthcare practice in a timely manner			
1	How can we use cardiovascular imaging to avoid invasive procedures?	How can we use cardiovascular imaging to make care pathways more efficient and improve the cost effectiveness of cardiovascular imaging?	For cardiovascular imaging AI techniques, how do we identify and reduce bias, improve generalisability and explain the results explainable AI?
2	How do we use cardiovascular imaging to guide management, reduce disease progression and improve prognosis for patients with coronary artery disease?	How can cardiovascular imaging be used to make more rapid and accurate diagnoses?	How do we create a national, representative, large scale cardiovascular imaging research database with ground truth annotation to enable training and validation of AI techniques?
3	How can cardiovascular imaging be used to make more rapid and accurate diagnoses?	How can we simplify, shorten and standardise cardiovascular image acquisition protocols for easier widespread use and reduced variability?	How can we simplify, shorten and standardise cardiovascular image acquisition protocols for easier widespread use and reduced variability?
4	How do we ensure patients have equal access to cardiovascular imaging when it is needed?	How can we use cardiovascular imaging to avoid invasive procedures?	Can we use AI to prioritise cardiovascular imaging scans for reporting and improve clinical decision-making based on cardiovascular imaging?
5	How can we use cardiovascular imaging to make care pathways more efficient and improve the cost effectiveness of cardiovascular imaging?	How do we link cardiovascular imaging data to other health data, for example, NHS patient records in a safe, secure, and responsible manner, and manage public trust when using unconsented data?	How do we link cardiovascular imaging data to other health data, for example, NHS patient records in a safe, secure, and responsible manner, and manage public trust when using unconsented data?

Green indicates that the question featured in the top five list for all three stakeholder groups for that prioritisation domain, orange indicates the question featured in two stakeholder groups and red only in one stakeholder group.

AI, artificial intelligence; NHS, National Health Service.

The top 10 questions for the 3 domains broadly overlapped with the most important areas of research identified in the survey of patients and the public. For the top 10 research questions ranked based on their positive impact for patients, 2 related to treating disease, 2 related to improving quality of life of patients and 6 related to diagnosing the disease.

### Phase 3: patient and public workshop

Members of the BHF Data Science Centre Public Advisory Group discussed these findings and highlighted the importance of addressing research questions that could be implemented today, as well as those that would facilitate new areas of treatment in the future. They discussed the need for both broad research questions and specific research questions, which would capture the nuances of their individual experiences. It was felt that patients would generally prioritise research based on what impacted them individually. Suggested methods to facilitate increased involvement of the public in future surveys included increasing the range of places that survey information was available, such as making it available in general practice surgeries. They also recommended using focus groups and individual interviews, to explore topics more deeply and gather more detailed feedback. Lastly, the group emphasised the need to target specific under-represented audiences. They recognised that certain groups, such as those from low-income backgrounds or those with disabilities, may be less likely to participate in surveys. To overcome this, they suggested tailoring the survey to specific groups and ensuring that the survey was accessible and easy to complete.

## Discussion

Using a modified Delphi technique with two rounds of prioritisation, we have established priorities for cardiovascular imaging research involving input from a wide range of stakeholders, including patients and the public and healthcare professionals. This consensus survey methodology enables a very large number of potential research questions to be focused into a prioritised list based on their positive impact for patients, potential to reduce inequalities in healthcare and ability to be implemented into the UK healthcare practice in a timely manner. Similarities and differences exist in the opinions of different stakeholder groups. We have ensured that the views of patients and the public were key drivers in shaping this process and their views were well represented.

This prioritisation exercise collected research questions from a wide range of stakeholders. Previous prioritisation exercises in this area have focused on research questions submitted by healthcare professionals or researchers, and so may be biased towards their areas of interest. In this survey, we included research questions submitted by patients and the public so that the results would represent views of the broader population. In addition, we used input from patients and the public to select the three domains for prioritisation in round two of the prioritisation. There are many domains that research questions can be prioritised on, including importance, impact, translation, economics, feasibility and potential to reduce inequalities. By using the domains that reflected the views of patents and the public, we believe we have created a relevant and representative list of research questions.

Previous prioritisation exercises have often focused on one disease or specific imaging modality.^4 6–11^ A survey of members of the British Society of Cardiovascular Imaging prioritised primarily research questions regarding CT.^4^ A broader survey on priorities in cardiovascular disease research by the European Research Area Network on Cardiovascular Diseases identified that earlier recognition of cardiovascular disease was the most important priority.^12^ To be most useful to our imaging research community, we incorporated all types of cardiovascular disease, including cardiac, vascular and neurovascular diseases. In addition, we ensured that the research questions were edited to be independent of particular imaging modalities. This did not mean we excluded questions that compared imaging types, as this is relevant for many of the highest rated research questions, but these comparisons were phrased generically rather than specifically. This ensures that these prioritised lists of research questions will be equally valuable for researchers focusing on MR, CT, nuclear medicine, echocardiography, electrocardiography and other imaging types. It also means that these prioritised research question lists will remain relevant and ‘modality agnostic’ should new imaging tests be developed.

The results of the public survey on how cardiovascular disease research should be prioritised will have impact beyond the field of imaging. All the research questions were rated as important to some degree, but patients and the public rated improving treatment, quality of life and diagnosis as the most important research questions. Interestingly, reducing the impact of healthcare on the environment was rated lowest, possibly because the impact of healthcare on the environment is not widely known, or because the other survey options which could have an immediate personal impact were rated higher. In terms of how research should be prioritised the highest rated domains were impact, translation, importance and reducing inequalities. Research that focused on the impact on the environment or society was again rated the lowest. This information can be used to help design cardiovascular research studies, beyond studies that involve cardiovascular imaging.

Different research questions featured in the highest rated questions for each of the three stake-holder groups (healthcare professionals, cardiovascular imaging researchers, data science and computer science researchers). Healthcare professionals gave higher ratings to questions that focused on identifying disease and guiding management. Cardiovascular imaging researchers gave higher ratings to questions that focused on identifying disease subgroups and improving imaging tests. Data science and computer science researchers gave higher ratings to questions that involved artificial intelligence and data curation, storage and linkage. These differences in opinion between different stakeholder groups are important to take into consideration. Nevertheless, there were also important similarities between the stakeholder groups, including the improvement of patient access to imaging tests and conducting research with a focus on the patient experience.

This study has some limitations which should be acknowledged. First, the survey participants were all based in the UK so extrapolation to other healthcare systems and populations should be undertaken cautiously. In particular, topics of importance in low-income countries will not be represented. Future surveys should consider research questions in other healthcare systems. Second, increasing the number of survey participants and number of rounds of prioritisation would further improve the accuracy of the prioritisation. It is likely that public participants already had an interest in cardiovascular diseases, and this may have impacted results. Third, not all submitted questions were included in this prioritisation exercise as this would be burdensome on survey participants. Nevertheless, the steering committee endeavoured to include questions representing as many of the topics as possible from the submitted questions. Fourth, the public survey involved a small subset of the population which may be biased to particular demographic groups. The subsequent workshop has helped with suggestions to engage an even broader audience for future surveys.

In conclusion, we have established priorities for cardiovascular research, and cardiovascular imaging research in particular, incorporating views of a diverse range of stakeholders, including patients and the public. These results could be used by researchers, funders and other organisations to help prioritise imaging research so that it has the greatest beneficial impact for patient care.

## Data Availability

No data are available.

## References

[1] WHO. Cardiovascular diseases (CVDs). 2021. Available: https://www.who.int/news-room/fact-sheets/detail/cardiovascular-diseases-(cvds) [Accessed 16 Aug 2021].

[2] BHF. UK factsheet. 2021. Available: https://www.bhf.org.uk/what-we-do/our-research/heart-statistics [Accessed 16 Aug 2021].

[3] Weir-McCall JR, Williams MC, Shah ASV, et al. National trends in coronary artery disease imaging: associations with health care outcomes and costs. JACC Cardiovasc Imaging 2023;16:659–71. 10.1016/j.jcmg.2022.10.022 36752441

[4] Yong GL, Weir-McCall J, Wilson M, et al. Research priorities in cardiovascular imaging. Open Heart 2020;7:e001389. 10.1136/openhrt-2020-001389 33046593 PMC7552921

[5] BHF, Data Science Centre. Workshop report - how can we use imaging data to better understand cardiovaascular disease? Zenodo, 2022. 10.5281/zenodo.6908183

[6] Drury NE, Herd CP, Biglino G, et al. Research priorities in children and adults with congenital heart disease: a James LIND alliance priority setting partnership. Open Heart 2022;9:e002147. 10.1136/openhrt-2022-002147 36600635 PMC9843188

[7] Taylor CJ, Huntley AL, Burden J, et al. Research priorities in advanced heart failure: James LIND alliance priority setting partnership. Open Heart 2020;7:e001258. 10.1136/openhrt-2020-001258 32606070 PMC7328807

[8] Drury NE, Stoll VM, Bond CJ, et al. Research priorities in single-ventricle heart conditions: a United Kingdom national study. Cardiol Young 2019;29:303–9. 10.1017/S104795111800224X 30572973 PMC6435189

[9] Orr JE, Ayappa I, Eckert DJ, et al. Research priorities for patients with heart failure and central sleep apnea. an official American Thoracic Society research statement. Am J Respir Crit Care Med 2021;203:e11–24. 10.1164/rccm.202101-0190ST 33719931 PMC7958519

[10] Lai FY, Abbasciano RG, Tabberer B, et al. Identifying research priorities in cardiac surgery: a report from the James LIND alliance priority setting partnership in adult heart surgery. BMJ Open 2020;10:e038001. 10.1136/bmjopen-2020-038001 PMC7473615 32883735

[11] Vascular Society of Great Britain and Ireland Peripheral Arterial Disease Special Interest Group James Lind Alliance Priority Setting Partnership, Pymer S, Harwood A, et al. Research priorities for patients with peripheral arterial disease: a James LIND alliance priority setting partnership. JVascSocGBIrel 2021;1:23–9. 10.54522/jvsgbi.2022.011

[12] ERA-CVD. Strategic research agenda for cardiovascular disease (SRA-CVD) - prioritisation consultation from the European research area network on cardiovascular diseases. 2019. Available: https://www.era-cvd.eu/media/content/SRA%20CVD%20consultation%20report_January_2020.pdf2023

