# Supplementary material for: Cardiovascular imaging research priorities
Source: Open Heart. 2023 Aug 16;10(2):e002378. doi: 10.1136/openhrt-2023-002378 (PMC10432634; doi:10.1136/openhrt-2023-002378)
Supplement: Supplementary data [file openhrt-2023-002378supp001.pdf]

Cardiovascular Imaging Research Priorities

Supplementary information

Supplementary Figure 1: Demographic characteristics of participants in the patient and public survey including (a) age, (b) gender, and (c) ethnicity.

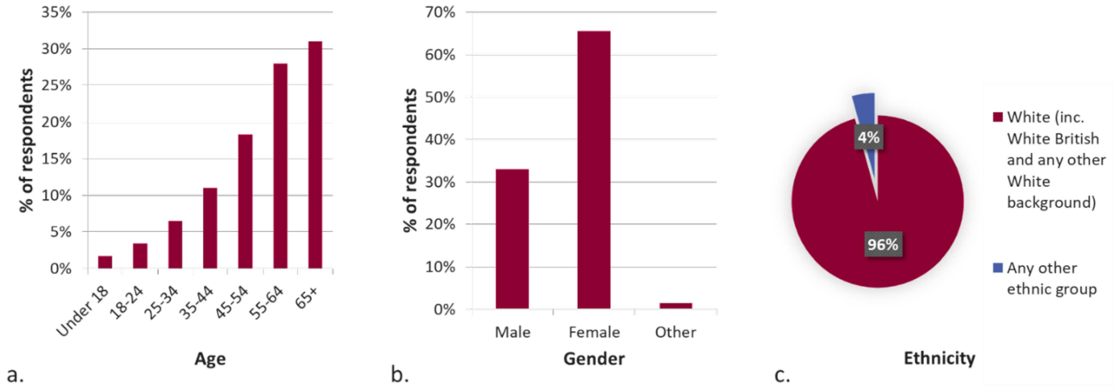

MacArthur JAL, *et al. Open Heart* 2024; 10:e002378. doi: 10.1136/openhrt-2023-002378

Supplementary Table 1: The 100 research questions selected for prioritisation in phase 2, ordered by their mean rating in the first round of prioritisation based on their positive impact for patients.

Question	Rating
How can we use cardiovascular imaging to avoid invasive procedures?	7.3 ± 1.7
Can cardiovascular imaging be used to identify vulnerable atherosclerotic plaques which could cause subsequent myocardial infarction?	7.3 ± 1.6
Can we use cardiac imaging to predict which patients with heart failure would benefit from different treatments?	7.2 ± 1.3
How can cardiovascular imaging be used to make more rapid and accurate diagnoses?	7.1 ± 1.7
How do we use cardiovascular imaging to screen for coronary artery disease, guide management, and prevent subsequent cardiovascular events?	7.1 ± 1.7
How do we use cardiovascular imaging to guide management, reduce disease progression and improve prognosis in coronary artery disease?	7.1 ± 1.8
Can we use cardiac imaging to predict which patients with coronary artery disease would benefit from more aggressive secondary prevention therapy in order to reduce the risk of myocardial infarction?	7.0 ± 1.7
How do we use cardiovascular imaging as an end-point in research trials (e.g. demonstrating response to treatment)?	6.9 ± 1.6
How do we link cardiovascular imaging data to other health data (e.g. NHS patient records) in a safe, secure and responsible manner?	6.9 ± 2.0
Can we use cardiovascular imaging to better diagnose the cause and subtypes of heart failure?	6.9 ± 1.3
Can we use cardiovascular imaging to target which patients with acute coronary syndromes would benefit from invasive management at an early timepoint?	6.9 ± 1.7
How do we use cardiovascular imaging to identify patients at risk of sudden cardiac death?	6.8 ± 1.7
How do we create a national, representative, large scale cardiovascular imaging research database with ground truth annotation to enable training and validation of AI techniques?	6.8 ± 2.1
How do we ensure patients have equal access to cardiovascular imaging when it is needed?	6.7 ± 2.2
How can we use cardiovascular imaging to make care pathways more efficient?	6.7 ± 2.0

MacArthur JAL, *et al. Open Heart* 2024; 10:e002378. doi: 10.1136/openhrt-2023-002378

Can we use cardiac imaging to better identify and characterise patients with cardiomyopathies?	6.6 ± 1.6
Can we use cardiovascular imaging to understand cardiovascular disease mechanisms?	6.6 ± 1.7
How can we better quantify cardiac ischaemia?	6.6 ± 2.0
Can we use AI to improve clinical decision making based on cardiovascular imaging?	6.6 ± 1.8
Can we use cardiovascular imaging to identify patients at risk of future stroke?	6.6 ± 1.4
Can we use cardiac imaging to predict which patients with inherited cardiac conditions or cardiomyopathies benefit from implanted cardiac devices?	6.6 ± 1.7
How do we use cardiac imaging to assess patients with myocardial infarction and non-obstructive coronary arteries (MINOCA)?	6.6 ± 1.7
How do we use cardiovascular imaging to identify people at risk of developing heart failure?	6.6 ± 1.7
How do we improve the cost effectiveness of cardiovascular imaging?	6.6 ± 1.9
How do we use cardiovascular imaging to improve the diagnosis of chest pain in women?	6.6 ± 1.8
How do we use cardiovascular imaging to detect cardiotoxicity from cancer therapy?	6.5 ± 1.5
Can we reduce the number of cardiac imaging tests that patients need during follow-up?	6.5 ± 1.8
Can we use AI to improve cardiovascular image acquisition, improve image quality, reduce radiation and contrast doses, and reduce motion artefacts?	6.5 ± 1.9
Which is more important in coronary artery disease - ischaemia or coronary stenosis on cardiac imaging?	6.5 ± 1.9
How can we simplify, shorten, and standardise cardiac MRI acquisition protocols for easier widespread use?	6.5 ± 1.9
How do we better train staff to perform and report cardiovascular imaging?	6.5 ± 2.0
Can we use cardiac imaging to better identify, stratify and follow-up patients with valvular heart disease and guide management?	6.4 ± 1.6
How do we use cardiac imaging to predict the risk of developing heart valve disease and assess its natural history?	6.4 ± 1.7
How can we use cardiovascular imaging to better identify and predict growth of aortic aneurysms and risk of dissection?	6.4 ± 1.8
How can we use cardiovascular imaging to understand the mechanisms and better diagnose acute aortic syndromes?	6.4 ± 1.5
Can we develop better ways to identify myocardial inflammation and infection?	6.4 ± 1.7
What is the best way to assess risk of future cardiovascular events in patients with valvular heart disease?	6.3 ± 1.7

Can cardiovascular imaging be used to screen asymptomatic patents and change management to prevent future events?	6.3 ± 2.0
What is the best way of rapidly integrating new imaging biomarkers and AI techniques into clinical practice?	6.3 ± 2.0
Can we use cardiovascular imaging to better understand the overlap between cardiovascular and other diseases (e.g., respiratory, kidney, and inflammatory diseases)?	6.3 ± 1.6
Can we use AI to prioritise cardiovascular imaging scans for reporting?	6.3 ± 1.7
Can we use cardiac imaging to assess which patients with atrial fibrillation are at risk of subsequent cardiovascular events?	6.3 ± 1.7
Can cardiac imaging be used to identify patients most likely to benefit from new expensive anti-inflammatory/lipid lowering therapies?	6.3 ± 1.8
How do we understand what information a cardiovascular imaging AI is using and how do we explain its results (explainable AI)?	6.3 ± 1.9
Can we use cardiovascular imaging to improve pre-operative risk stratification?	6.3 ± 1.8
Is the diagnostic accuracy of cardiovascular imaging tests affected by gender or ethnicity?	6.3 ± 1.8
How can we use cardiovascular imaging to better identify cardiac device infection?	6.2 ± 1.8
Can we work out which patients do not derive benefit from cardiovascular imaging?	6.2 ± 2.0
Can we use cardiac imaging to understand whether inflammation is an important predictor of atheroma?	6.2 ± 1.8
Can we use imaging to monitor changes in the heart with diabetes mellitus, and the impact of treatments on these changes?	6.2 ± 1.9
How do we identify and reduce bias in cardiovascular imaging AI techniques (e.g., secondary to gender, ethnicity, preconceived ideas)?	6.2 ± 2.2
Can we use cardiovascular imaging to better understand complex congenital heart disease and guide management?	6.1 ± 1.8
What is the effect of image acquisition variability (i.e., different scanners or protocols) on the accuracy and generalisation of AI techniques and how do we standardise this?	6.1 ± 2.0
What is the best process to ensure that clinically important non-cardiac findings on cardiac imaging are not missed?	6.1 ± 2.0
What is the best method to assess and follow up cardiac function in patients with transient heart failure e.g., oncology, sepsis?	6.1 ± 1.7
How can we use cardiovascular imaging to understand the link between cardiovascular and neurodegenerative diseases?	6.1 ± 2.0
Can we use cardiac imaging to understand changes in the heart after medical treatment or surgery?	6.1 ± 1.9
Can we use cardiac imaging to predict which patients with atrial fibrillation would benefit from different treatments (such as medical therapy, ablation, anticoagulation, device therapy)?	6.0 ± 1.8
How can we improve the assessment of cardiac fibrosis?	6.0 ± 1.7

MacArthur JAL, *et al. Open Heart* 2024; 10:e002378. doi: 10.1136/openhrt-2023-002378

How do we better manage data storage; anonymisation and access for cardiovascular imaging research?	6.0 ± 2.2
How do we improve how we communicate the results of cardiovascular imaging to patients?	6.0 ± 2.0
Can we use virtual TAVI implantation on CT to assess potential complications pre-operatively?	6.0 ± 1.8
How do changes in cardiac metabolism in early heart failure translate to changes in cardiac function?	6.0 ± 1.9
Can we use cardiac imaging to predict which patients are at risk of developing atrial fibrillation?	6.0 ± 1.8
What is the optimum cut-off value for intervention on a thoracic or abdominal aortic aneurysm?	6.0 ± 2.0
Can we use cardiovascular imaging to understand genetic predispositions for cardiovascular disease?	6.0 ± 1.7
How do we standardise cardiovascular imaging acquisition across manufacturers?	5.9 ± 2.1
Can we use cardiovascular imaging to better select patients for participation in research studies (e.g., those more likely to benefit from new drugs)?	5.9 ± 1.8
Can cardiovascular imaging be used to identify the source of cryptogenic stroke?	5.9 ± 1.6
How can we reduce or avoid the use of radiation in cardiovascular imaging?	5.9 ± 2.2
How do we use cardiovascular imagining to understand the pathophysiology of atrial fibrillation?	5.9 ± 1.8
What is the impact of CTFFR on outcomes for patients with coronary artery disease?	5.9 ± 2.5
How do we manage public trust when using unconsented data in cardiovascular imaging research?	5.9 ± 2.1
What is the impact of valve intervention (surgery, TAVI) on cardiac imaging indices and how does valve function change during follow-up?	5.8 ± 1.9
How do we use cardiovascular imaging to understand age related changes in the shape and function of the heart, brain and blood vessels?	5.8 ± 1.8
How can automated phenotypes be derived from cardiovascular imaging at scale?	5.8 ± 1.9
Can we use cardiovascular imaging to improve the design of cardiac devices (e.g., closure devices for atrial septal defects)?	5.7 ± 1.8
Can we use cardiovascular imaging to understand the mechanisms underlying the metabolic syndrome?	5.7 ± 1.9
How can we use cardiac imaging to understand the pathology underlying asymptomatic ECG abnormalities?	5.7 ± 1.7
Can non-cardiovascular imaging be used to identify cardiovascular disease?	5.7 ± 2.0
How do we improve segmentation of anatomical structures on cardiovascular imaging?	5.7 ± 2.2

MacArthur JAL, *et al. Open Heart* 2024; 10:e002378. doi: 10.1136/openhrt-2023-002378

How can we use cardiovascular imaging to understand the influence of the environment, sociodemographic and economic factors on cardiac structure and function?	5.7 ± 1.9
Can we use cardiovascular imaging to identify autonomic cardiac dysfunction?	5.6 ± 1.9
Which patients benefit from screening for abdominal aortic aneurysms?	5.6 ± 1.6
How can we use natural language processing to improve cardiovascular imaging AI research?	5.6 ± 2.2
What is the optimal number of images needed to develop and test a new cardiovascular imaging AI technique?	5.6 ± 2.2
How can we use imaging to identify neurological changes such as those associated with vascular dementia?	5.6 ± 1.9
How do we use cardiovascular imaging to understand the overlap between the heart and the brain (the heart-brain axis)?	5.5 ± 1.9
How are imaging resources being used across the UK?	5.5 ± 2.0
How do we make cardiovascular imaging more comfortable, less painful and less frightening for patients?	5.5 ± 2.1
How can we better use cardiovascular imaging in utero to diagnose congenital heart disease?	5.4 ± 1.9
Can we use cardiovascular imaging to help create digital twins for future research?	5.4 ± 2.1
How do we use cardiovascular imaging to better guide management of peripheral vascular disease?	5.3 ± 1.8
How can we use retinal imaging to identify cardiovascular disease and risk of cardiovascular events?	5.3 ± 1.9
How can we use cardiovascular imaging to assess the impact of COVID-19 on the cardiovascular system?	5.3 ± 2.3
How can we reduce the environmental impact of cardiovascular imaging?	5.1 ± 2.3
Can we use cardiac imaging to assess angiogenesis?	5.1 ± 2.0
Can we use cardiovascular imaging to better understand differences in cardiac structure and function in athletes?	5.0 ± 2.1
What is the incidence of bicuspid aortic valve disease and its natural history in a contemporary UK population?	4.7 ± 1.9
What is the impact of COVID-19 on cardiovascular imaging services?	4.5 ± 2.3

Mean ± standard deviation.

AI, artificial intelligence; CT, computed tomography; COVID-19, coronavirus disease 2019; ECG, electrocardiogram; FFR, fractional flow reserve; MRI, magnetic resonance

imaging; NHS, National health service; TAVI, transcatheter aortic valve implantation; UK, United Kingdom.

Supplementary Table 2: The 32 research questions selected for prioritisation in phase 3, ordered by their mean rating in the second round of prioritisation based on their positive impact for patients.

Question	Rating
How do we ensure patients have equal access to cardiovascular imaging when it is needed?	7.6±1.6
How can cardiovascular imaging be used to make more rapid and accurate diagnoses?	7.5±1.4
How do we use cardiovascular imaging to guide management, reduce disease progression, and improve prognosis for patients with coronary artery disease?	7.3±1.4
How can we use cardiovascular imaging to avoid invasive procedures?	7.2±1.7
How do we use cardiovascular imaging to identify patients at risk of sudden cardiac death?	7.0±1.5
Can we use cardiovascular imaging to better diagnose the cause and subtypes of heart failure and predict which patients with heart failure would benefit from different treatments?	7.0±1.4
Can we reduce the number of cardiac imaging tests that patients need during follow up?	6.9±2.0
How do we use cardiovascular imaging to identify people at risk of developing heart failure?	6.9±1.4
How do we better train staff to perform and report cardiovascular imaging?	6.7±1.8
How do we link cardiovascular imaging data to other health data e.g. NHS patient records in a safe, secure and responsible manner, and manage public trust when using unconsented data?	6.6±2.0
What is the best way to assess risk of future cardiovascular events in patients with valvular heart disease?	6.6±1.4
How can we use cardiovascular imaging to make care pathways more efficient and improve the cost effectiveness of cardiovascular imaging?	6.6±1.7
Can we use cardiac imaging to better identify and characterise patients with cardiomyopathies and identify which patients benefit from implanted cardiac devices?	6.6±1.4
How can we use imaging to identify patents at risk of neurological conditions such as stroke or vascular dementia?	6.6±1.5

Can screening for coronary artery disease with cardiovascular imaging be used to guide management and improve outcomes in the general population or in the pre-operative setting?	6.5±1.5
How can we simplify, shorten, and standardise cardiovascular image acquisition protocols for easier widespread use and reduced variability?	6.5±1.7
How do we use cardiovascular imaging to detect cardiotoxicity from cancer therapy?	6.5±1.5
Can we use AI to prioritise cardiovascular imaging scans for reporting and improve clinical decision making based on cardiovascular imaging?	6.5±1.7
Can we use AI to improve cardiovascular image acquisition, improve image quality, reduce radiation and contrast doses, and reduce motion artefacts?	6.4±1.6
How can we use cardiovascular imaging to better identify and predict growth of aortic aneurysms and risk of dissection?	6.3±1.6
How do we create a national, representative, large scale cardiovascular imaging research database with ground truth annotation to enable training and validation of AI techniques?	6.3±2.2
What is the best way of rapidly integrating new imaging biomarkers and AI techniques into clinical practice?	6.2±2.0
Can we develop better ways to identify myocardial inflammation infection, and fibrosis and cardiac device infection?	6.1±1.6
Can we use cardiovascular imaging to understand cardiovascular disease mechanisms?	6.1±1.8
How do we use cardiovascular imaging to select patients for participation in research studies (e.g., those more likely to benefit from new drugs) or as an end point in research trials (e.g., demonstrate response to treatment)?	6.1±1.9
Can we use cardiovascular imaging to better understand the overlap between cardiovascular and other diseases e.g., respiratory, kidney, inflammatory, and neurodegenerative diseases?	6.1±1.4
Can we use cardiac imaging to predict which patients are at risk of developing atrial fibrillation?	6.0±1.7
For cardiovascular imaging AI techniques, how do we identify and reduce bias, improve generalisability, and explain the results explainable AI?	6.0±1.9
How do we use cardiovascular imaging to understand age related changes in the shape and function of the heart, brain, and blood vessels?	5.8±1.7
How do we better manage data storage, anonymisation and access for cardiovascular imaging research?	5.7±2.1

MacArthur JAL, *et al. Open Heart* 2024; 10:e002378. doi: 10.1136/openhrt-2023-002378

How can we use cardiovascular imaging to understand the influence of the environment, sociodemographic, and economic factors on cardiac structure and function?	5.6±1.9
Can we use cardiovascular imaging to identify autonomic cardiac dysfunction?	5.1±1.7

Mean ± standard deviation.

AI, artificial intelligence.

MacArthur JAL, *et al. Open Heart* 2024; 10:e002378. doi: 10.1136/openhrt-2023-002378

Supplementary Information: We gratefully acknowledge the time and effort of the survey participants, including the following who consented to their inclusion in this list.

Name	Affiliation
Adam Hartley	Imperial College London
Alastair Moss	University of Leicester
Alejandro Frangi	University of Leeds
Alexander Wilson	Barts Health NHS Trust
Alistair Young	King's College London
Andrew Archbold	Barts Heart Centre
Anish Bhuva	Institute of Health Informatics, UCL
Anna Beattie	The Newcastle Hospitals NHS Trust
Bilal Kirmani	Liverpool Heart and Chest Hospital
Brian Morrissey	NHS Grampian; University of Aberdeen
Christian Camm	University of Oxford
Chrysoula Fragkiadaki	-
David Buckley	University of Leeds
Emily King	Swansea Bay University Healthboard
Emma Lane	University of Portsmouth
Emmanouela Kampouraki	Leeds Teaching Hospitals Trust
Esther Puyol-Antón	King's College London
Gavin Murphy	University of Leicester
Gerry McCann	University of Leicester and NIHR Leicester Biomedical Research Centre
Gez Williams	University of Leicester
Guy Lloyd	Barts Heart Centre; Queen Marys University of London; University College London; Cleveland Clinic London
James Hobkirk	The University of Hull
Jason Ali	Papworth Hospital, Department of Cardiothoracic Surgery
Jasper Boeddinghaus	Centre for Cardiovascular Research Edinburgh & Cardiovascular Research Institute Basel (CRIB)
John Gierula	University of Leeds
Jonathan Rodrigues	Royal United Hospital Bath, Department of Radiology
Jonathan Weir-McCall	University of Cambridge
Joseph Jacob	University College London Respiratory and Centre for Medical Image Computing
Jun Cheong	-
Kang-Ling Wang	University of Edinburgh
Kathryn Griffin	University of Leeds
Krishnaraj Rathod	Barts Heart Centre
Marian Pop	George Emil Palade University of Medicine
Mark Harbinson	Queen's University Belfast

Name	Affiliation
Markos Klonizakis	Lifestyle Exercise and Nutrition Improvement (LENI) Research Group, Sheffield Hallam University
Massimo Caputo	University of Bristol
Matthew Shun-Shin	Imperial College London
Michael Mcdermott	University of Edinburgh
Michelle Williams	British Heart Foundation Data Science Centre
Neil Bodagh	King's College London
Nik Sabharwal	Oxford Heart Centre
Nikesh Jathanna	Department of Cardiology
Omar Asghar	-
Parthiban Arumugam	Manchester NHS Foundation Trust
Phinehas Arkorful	Cancer Research UK
Ramya Dhandapani	Sandwell and West Birmingham Hospitals NHS Trust
Rick Southworth	King's College London
Ricky Vaja	Imperial College London
Robert Cronshaw	Royal Infirmary of Edinburgh
Ryan Grech	NHS Greater Glasgow and Clyde
S Richard Underwood	Royal Brompton & Harefield Hospitals
Samer Alabed	NIHR Clinical Lecturer in Radiology
Simon Corbett	University Hospital Southampton NHS Foundation Trust
Sofia S. Villar	MRC Biostatistics Unit, University of Cambridge
Sree Kondapally	Cardiovascular Clinical Academic Group
Stefan Neubauer	Division of Cardiovascular Medicine
Steffen Petersen	Queen Mary University of London and Barts Health NHS Trust
Stelios Iacovides	-
Sujoy Roy	-
Sunil Nair	-
Theodora Zaglavara	Interbalkan European Medical Center
Tom Johnson	University of Bristol
Vincent McCaughan	Barts Health Trust
William Topping	-
Yvonne Jones	-

MacArthur JAL, *et al. Open Heart* 2024; 10:e002378. doi: 10.1136/openhrt-2023-002378
